# Self-Calibration for Star Sensors

**DOI:** 10.3390/s24113698

**Published:** 2024-06-06

**Authors:** Jingneng Fu, Ling Lin, Qiang Li

**Affiliations:** 1 Institute of Optics and Electronics, Chinese Academy of Sciences, Chengdu 610042, China; linling_yj@163.com (L.L.); liqiang@ioe.ac.cn (Q.L.); 2 Key Laboratory of Science and Technology on Space Optoelectronic Precision Measurement, Chinese Academy of Sciences, Chengdu 610042, China; 3 University of Chinese Academy of Sciences, Beijing 100049, China; 4 Youth Innovation Promotion Association, Chinese Academy of Sciences, Beijing 100049, China

**Keywords:** star sensor, camera calibration, self-calibration, on-orbit calibration, interstar angle invariance, constant optical path constraint

## Abstract

Aiming to address the chicken-and-egg problem in star identification and the intrinsic parameter determination processes of on-orbit star sensors, this study proposes an on-orbit self-calibration method for star sensors that does not depend on star identification. First, the self-calibration equations of a star sensor are derived based on the invariance of the interstar angle of a star pair between image frames, without any requirements for the true value of the interstar angle of the star pair. Then, a constant constraint of the optical path from the star spot to the center of the star sensor optical system is defined to reduce the biased estimation in self-calibration. Finally, a scaled nonlinear least square method is developed to solve the self-calibration equations, thus accelerating iteration convergence. Our simulation and analysis results show that the bias of the focal length estimation in on-orbit self-calibration with a constraint is two orders of magnitude smaller than that in on-orbit self-calibration without a constraint. In addition, it is shown that convergence can be achieved in 10 iterations when the scaled nonlinear least square method is used to solve the self-calibration equations. The calibrated intrinsic parameters obtained by the proposed method can be directly used in traditional star map identification methods.

## 1. Introduction

A star sensor represents an important component of high-precision satellite attitude calculations and control systems. To determine an attitude value through star identification, it is necessary to calibrate each star sensor in a laboratory first. However, the harsh launching process of spacecraft and the complex physical environment in space will inevitably affect a calibrated star sensor and make it deviate from its laboratory-calibrated state. When the intrinsic parameters of a star sensor deviate obviously from its laboratory-calibrated values, it is necessary to perform on-orbit calibration to prevent a decrease in the attitude measurement accuracy of the star sensor. Therefore, on-orbit calibration is very important for high-precision star sensors.

At present, the on-orbit calibration methods for star sensors can be roughly divided into two types: attitude-independent methods and attitude-dependent methods [1,2]. Attitude-dependent on-orbit calibration methods require prior information on a star sensor’s attitude, which is typically obtained by gyros [1] or landmarks [3]. However, the coupling of the extrinsic and intrinsic parameters of a star sensor inevitably causes these parameters to affect each other. In contrast, attitude-independent on-orbit calibration methods only make use of the invariance of the interstar angle of a star pair between the object space and the image space of a star sensor and thus do not require information on the star sensor’s attitude. This approach avoids the coupling between the extrinsic and intrinsic parameters of a star sensor. Accordingly, most studies on the on-orbit calibration algorithms of star sensors have focused on attitude-independent methods. These attitude-independent methods can be further divided into batch processing algorithms [4,5] and sequential processing algorithms [6,7] according to the characteristics of the data used for processing. Batch processing algorithms extract the star spot information from multiple image frames at the same time and, therefore, require a relatively large database to store the image plane coordinates of multiple frames and celestial coordinate information. However, these algorithms are unlikely to overfit their parameters due to the comprehensiveness of the information used in the calculation process. Sequential processing algorithms employ the Kalman filter and use one frame of data at once to update the star sensors’ intrinsic parameters and, thus, have a low requirement for data storage but are very sensitive to the setting of filter parameters and tend to overfit the current star pattern. The implementation of attitude-independent calibration methods is based on the premise that the relationship between star spots and guide stars has been established, that is, star identification is completed before the intrinsic parameters of the star sensor are determined; these methods are also called dimensionless (or non-calibration) star map identification algorithms [8,9]. Unlike traditional star map identification algorithms, where the minimum identifiable star map is a star pair [10], dimensionless star map identification algorithms take a star triangle as the minimum identifiable star map. A disadvantage of these algorithms is the requirement for a large database and complex data retrieval algorithm. For instance, the spherical triangle star map identification algorithm proposed in [9] requires a database of nearly 100 MB, as well as a high-dimensional k-vector retrieval algorithm [11].

Since a star sensor is a special type of camera, theoretically, the calibration methods for common cameras can be used for star sensor calibration. Camera calibration methods can be roughly divided into two types: traditional calibration methods [12] and self-calibration methods [13,14,15,16,17,18]. Traditional calibration methods rely on the high geometric precision of one-, two-, or three-dimensional objects to establish object–image equations and then solve the intrinsic parameters of the camera. These methods include the traditional on-orbit calibration method of star sensors. Self-calibration methods only make use of the corresponding relationship between multiple images, namely, they rely only on the information in an image space. When a camera self-calibration method is applied to the on-orbit calibration of the intrinsic parameters of a star sensor, the chicken-and-egg problem of star identification and intrinsic parameter determination can be avoided, which means that star sensor calibration can be accomplished before the star identification process. However, due to the irregular and uncorrelated distribution of vanishing points (i.e., star spots formed by parallel starlight) on the focal plane, regardless of whether a camera self-calibration method is based on the Kruppa equation [13,14], dual absolute quadric surface [15], a hierarchical step-by-step method [16,17], or Pollefeys’ modulus constraint [18], it cannot be directly used for the on-orbit calibration of star sensors.

To address the chicken-and-egg problem in the star identification and intrinsic parameter determination processes of on-orbit star sensors, this study proposes a self-calibration method that differs from previously proposed methods for star sensors. First, the intrinsic parameter equations of a star sensor are derived based on the invariance of the interstar angle of a star pair between image frames. Then, the constant constraint of an optical path from the star spot to the center of the star sensor optical system is defined to reduce the biased estimation in self-calibration. Finally, a scaled nonlinear least square method is developed to solve the derived equations to accelerate iteration convergence. Our simulation and analysis results show that convergence can be achieved in 10 iterations when the scaled nonlinear least square method is used to solve the self-calibration equations.

The remainder of this paper is organized as follows. Section 2 presents the classical model of star sensor on-orbit calibration based on the pinhole imaging model. Section 3 introduces an on-orbit self-calibration model with an optical path constraint. Section 4 describes the process of solving on-orbit self-calibration equations using the scaled nonlinear least square method. Section 5 describes the simulation and experimental results. Finally, Section 6 concludes this study.

## 2. The Classical On-Orbit Calibration Model

**Figure 1:**
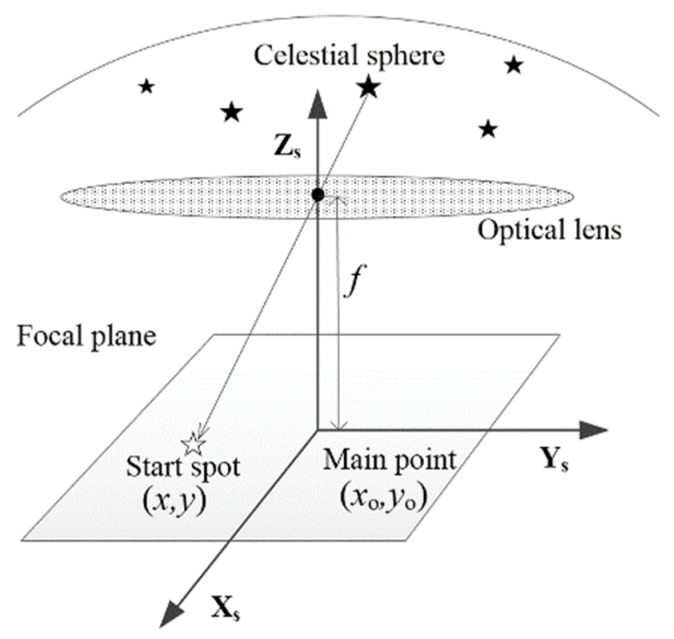
The pinhole imaging model of a star sensor.

This study simplifies the calibration of the intrinsic parameters of a star sensor. If the pinhole presented in Figure 1 is used as an imaging model of a star sensor, its intrinsic parameters include only the principal point and focal length, and the calibration of the principal point is equivalent to compensation of zero-order distortion [19]. For the compensation of higher-order distortion, please refer to the research presented in [4,20].

Assume that the right ascension and declination of the ith (1≤i≤N) star extracted from the observed image are $\left(\alpha_{i},\delta_{i}\right)$, respectively; then, its unit direction in the celestial sphere coordinate system is expressed as follows:

(1) $$v_{i}={\left(\cos\alpha_{i}\cos\delta_{i},\sin\alpha_{i}\cos\delta_{i},\sin\delta_{i}\right)}^{T}.$$

The image coordinates of the star in the corresponding star sensor coordinate system are $\left(x_{i},y_{i}\right)$, and the unit direction of the star in the star sensor coordinate system, under the assumption of ideal pinhole imaging, is defined by

(2) $$w_{i}=\frac{1}{\sqrt{{\left(x_{i}-x_{o}\right)}^{2}+{\left(y_{i}-y_{o}\right)}^{2}+f^{2}}}\left[\begin{array}{c} -\left(x_{i}-x_{o}\right)\\ -\left(y_{i}-y_{o}\right)\\ f \end{array}\right].$$

**Figure 2:**
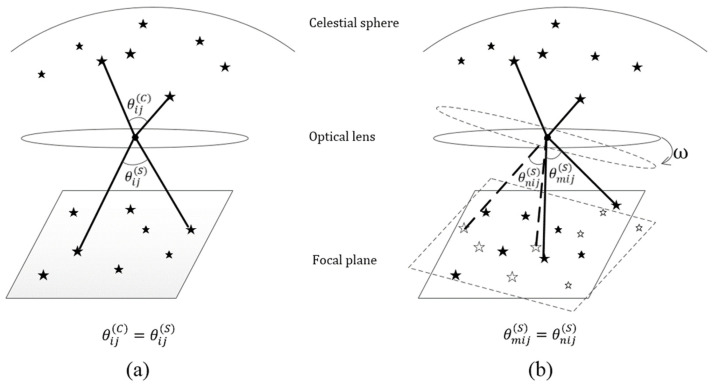
(**a**) Invariance of the interstar angle of a star pair in the object and image spaces, which represent the basis for traditional on-orbit calibration; (**b**) invariance of the interstar angle of a star pair in the image space at different times, which is the basis for on-orbit self-calibration.

Generally, the calibration methods for the intrinsic parameters of star sensors include two calibration algorithms: an algorithm relying on the information of a star sensor’s attitude and an algorithm not relying on this information, as shown in Figure 2a. These two algorithms are defined as follows:

(3) $$M_{IS}w_{i}-v_{i}=0,\;\;1\leq i\leq N,$$

and

(4) $$w_{i}^{T}w_{j}-v_{i}^{T}v_{j}=0,\;\;1\leq i<j\leq N,$$

where $M_{IS}$ is the attitude matrix of a star sensor. 

Being independent of the attitude information of a star sensor means that the problem of coupling between the extrinsic and intrinsic parameters is avoided. For this reason, most studies on the on-orbit calibration algorithms of star sensors have focused on attitude-independent methods.

## 3. On-Orbit Self-Calibration Model

When a star sensor rotates, it tracks all the star spots (i.e., a total of $N_{S}$ star spots) in an image sequence with a length of $N_{F}$ and denotes the stars as $1,2,\ldots ,N_{S}$. Considering the invariance of the interstar angle of a star pair $\left(i,j\right)\left(1\leq i<j\leq N_{S}\right)$ in different image frames $\left(m,n\right)\left(1\leq m<n\leq N_{F}\right)$, the interstar angle of the ith and jth star spots in the star sensor coordinate system extracted from the image frame m equals the interstar angle of the ith and jth star spots in the star sensor coordinate system extracted from the image frame n; thus, the following equation can be derived (see Figure 2b):

(5) $$w_{mi}^{T}w_{mj}-w_{ni}^{T}w_{nj}=0,\;1\leq m<n\leq N_{F},\;1\leq i<j\leq N_{S}.$$

According to the pinhole imaging model, the unit direction of a star in a star sensor coordinate system is given by

(6) $$w_{mi}=\frac{1}{\sqrt{{\left(x_{mi}-x_{o}\right)}^{2}+{\left(y_{mi}-y_{o}\right)}^{2}+f^{2}}}\left[\begin{array}{c} -\left(x_{mi}-x_{o}\right)\\ -\left(y_{mi}-y_{o}\right)\\ f \end{array}\right],$$

where $\left(x_{mi},y_{mi}\right)$ are the image coordinates of the ith star spot extracted from the mth image frame.

**Figure 3:**
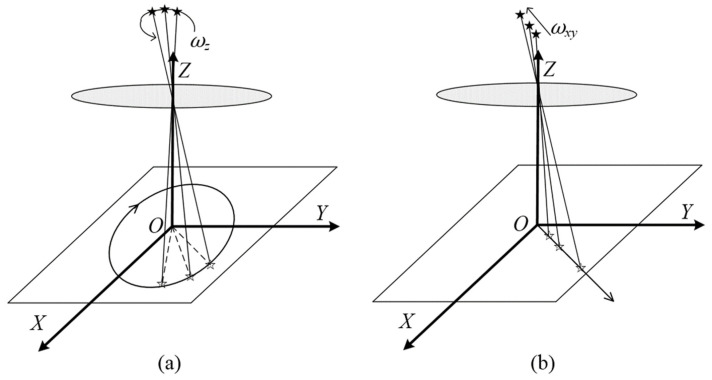
Illustration of the motion decomposition process of a star spot on the image plane: (**a**) rotation around the optical axis; (**b**) motion in the radial direction.

The motion of a star spot on the image plane can be decomposed into its rotation around the optical axis (refer to Figure 3a) and its radial motion (refer to Figure 3b) [21]. If star spots rotate only around the optical axis of the image plane, Equation (5) can be simplified to

(7) $$x_{o}\left(x_{mi}+x_{mj}-x_{ni}-x_{nj}\right)+y_{o}\left(y_{mi}+y_{mj}-y_{ni}-y_{nj}\right)=x_{mi}x_{mj}+y_{mi}y_{mj}-x_{ni}x_{nj}-y_{ni}y_{nj}.$$

Equation (7) is a linear equation of the principal point, which does not include the focal length. However, this equation is valid regardless of the focal length’s value. Therefore, the angular velocity of the radial motion of star spots on the image plane cannot be zero if the aim is to estimate their focal length.

Assume that the star spot extraction error obeys the Gaussian distribution of $\delta x_{mi},\delta y_{mi}\sim N\left(0,\frac{\sigma_{S}^{2}}{2}\right)$; then, when the star spot extraction error satisfies the condition of $\sigma_{S}>0$, the focal length estimation is larger than the true value of the focal length, regardless of whether the traditional on-orbit calibration model of Equation (4) or the on-orbit self-calibration model of Equation (5) is adopted. Further, considering the on-orbit self-calibration model of Equation (5), the following bias and variance tradeoff analysis can be made. When the focal length deviates from its true value, the direct contribution of the focal length bias to the total error of ($w_{mi}^{T}w_{mj}-w_{ni}^{T}w_{nj}$) increases. The direction error $\frac{\sigma_{S}}{f}$, converted from the star spot extraction error of $\sigma_{S}>0$, decreases with the focal length, which decreases the indirect contribution of the random error of star spot extraction to the total error of ($w_{mi}^{T}w_{mj}-w_{ni}^{T}w_{nj}$); this decrease exhibits a downward trend. When the total error of ($w_{mi}^{T}w_{mj}-w_{ni}^{T}w_{nj}$) reaches its minimum value, the corresponding estimated focal length is larger than the true focal length. A similar analysis could be made for the traditional on-orbit calibration model of Equation (4). However, since the on-orbit self-calibration model of Equation (5) is not constrained by the true value of the interstar angle of a star pair, it has a larger bias in its focal length estimation compared to the traditional on-orbit calibration model of Equation (4). 

To reduce the bias of the focal length estimation, it is necessary to define a focal length constraint. The simplest constraint is to assume that the focal length is a constant, but this is not conducive to solving Equations (4) and (5), so it is necessary to define a higher-order constraint on the focal length. Simulations have shown that when the optical path $r_{mi}=\sqrt{{\left(x_{mi}-x_{o}\right)}^{2}+{\left(y_{mi}-y_{o}\right)}^{2}+f^{2}}$ between the star spot on the image plane and the lens center of an optical system is a constant $C_{mi}$, the problem of the biased estimation in Equations (4) and (5) can be mitigated by establishing a mutual constraint between the coordinates of the star spot and the focal length.

The final form of the proposed on-orbit self-calibration model, independent of star identification, is defined as follows:

(8) $$w_{mi}^{T}w_{mj}-w_{ni}^{T}w_{nj}=0,\;\;1\leq m<n\leq N_{F},\;\;1\leq i<j\leq N_{S},$$

(9) $$s.t.\;r_{mi}=C_{mi},\;\;1\leq m\leq N_{F},\;\;1\leq i\leq N_{S}.$$

## 4. Solving Self-Calibration Equations

After defining the optical path constraint $r_{mi}=C_{mi}$, the derivative of $w_{mi}$, with respect to the intrinsic parameters, becomes more concise. However, the convergence speed of the nonlinear least square method [22] is slow, and the solution process requires numerous iterations. In view of that, this study introduces the convergence scale parameter λ to increase the convergence speed.

Denote the intrinsic parameter vector of a star sensor as $X={\left(x_{o},y_{o},f\right)}^{T}$ and solve Equations (8) and (9) iteratively using the scaled nonlinear least square method. Then, it holds that

(10) $$X^{\left(t+1\right)}=X^{\left(t\right)}+\lambda {\left(A^{T}A\right)}^{-1}A^{T}B$$

where t is the iteration number; λ is the convergence scale parameter, and λ>1; and the Jacobi matrix *A* and vector *B* are, respectively, defined as follows:

(11) $$A={\frac{\partial {\left(w_{11}^{T}w_{12}-w_{21}^{T}w_{22},\ldots ,w_{K-1,N_{F}-1}^{T}w_{K-1,N_{F}}-w_{K,N_{F}-1}^{T}w_{K,N_{F}}\right)}^{T}}{\partial X}|}_{X=X^{\left(t\right)}},$$

and

(12) $$B={{\left(w_{11}^{T}w_{12}-w_{21}^{T}w_{22},\ldots ,w_{K-1,N_{F}-1}^{T}w_{K-1,N_{F}}-w_{K,N_{F}-1}^{T}w_{K,N_{F}}\right)}^{T}|}_{X=X^{\left(t\right)}}.$$

Under the optical path constraint of $r_{mi}=C_{mi}$, the derivative of $w_{mi}$, with respect to the intrinsic parameter vector X, is expressed by

(13) $$\{\begin{array}{c} \frac{\partial w_{mi}}{\partial x_{o}}=\frac{{\left(1,0,0\right)}^{T}}{r_{mi}}\\ \frac{\partial w_{mi}}{\partial y_{o}}=\frac{{\left(0,1,0\right)}^{T}}{r_{mi}}\\ \frac{\partial w_{mi}}{\partial f}=\frac{{\left(0,0,1\right)}^{T}}{r_{mi}} \end{array}.$$

According to Equation (13) and $r_{mi}=C_{mi}$, $w_{mi}$ represents a linear function of the intrinsic parameters. However, it is a very interesting phenomenon that $C_{mi}$, which is not present explicitly in the equation to be solved, can indirectly change the convergence process and results of the self-calibration solution algorithm.

## 5. Simulation and Analysis Results

The proposed algorithm and several comparison algorithms were used in a simulation experiment conducted on a computer with two Intel Xeon Gold 5218R CPUs @2.1 GHz (with 40 cores in total) and 32 GB of memory. To improve the efficiency of the simulations, Matlab/C mixed programming was used for the test, and the parallel computing toolbox of Matlab was used for multi-core operations (two programs were opened at the same time, each program called for 16 cores). Compared to the scenario of using a single-core CPU and pure Matlab programming, the time needed to accomplish the simulation experiment was shortened by two orders of magnitude. The entire simulation experiment required the computer to run the test programs continuously for 17 days. 

The basic parameters of the star sensor used in the simulation experiment, originating from a real star sensor, were as follows: field of view (FOV): Φ=20°; pixel array: 1536 × 1536; position $\left(x_{o},y_{o}\right)$ of the theoretical principal point of the optical system: (768, 768); pixel size: 5.5 um × 5.5 um; focal length f: 24.4455 mm.

The star spots in the zeroth image frame $N_{S}$ were randomly distributed in a cone with a diameter of 10°. The first $N_{F}$ frames of the star sensor images were obtained by randomly rotating the star sensor, and $N_{S}$ star spots in each image frame were guaranteed to be present in the field of view of the star sensor.

The following four models were tested and compared in the simulation experiments:

1. A traditional calibration method without constraint (TCOC), as shown in Equation (4);
2. Traditional calibration methods with constraint (TCWC), as shown in Equations (4) and (9);
3. A self-calibration method without constraint (SCOC), as shown in Equation (8);
4. A self-calibration method with constraint (SCWC), as shown in Equations (8) and (9).

In the simulation experiments, different combinations of the values of the star spot extraction error $\sigma_{S}$, the number of image frames involved in the image frame number $N_{F}$, and the number of star spots per image $N_{S}$ were used. Particularly, star sensors used the centroid to determine the coordinates of the star spots on the focal plane, and the star point extraction error was generally $\sigma_{S}=0.01\;\mathrm{pixel}-0.50\;\mathrm{pixel}$ [23]. A star sensor with an FOV value of Φ = 20° operated normally at 5.5 Mv; the average and maximum star numbers were 25 and 80 in the FOV, respectively. Meanwhile, the average star number $N_{S}$ in a cone with a diameter of 10° was eight, which is too small for self-calibration, so it was necessary to increase the detection magnitude by increasing the exposure time or the gain in the self-calibration mode of the star sensor. When the detection magnitude increased by 1.0 Mv, the average and maximum star numbers in a cone with a diameter of 10° were 20 and 77, respectively. Based on the above analysis, the number of stars in each image of $N_{S}$ was set to a value in the range of 20–100. Furthermore, the labels of the stars $\left(i,j\right)\left(1\leq i<j\leq N_{S}\right)$ and the labels of the frames $\left(m,n\right)\left(1\leq m<n\leq N_{F}\right)$ obeyed the law of exchange in Equations (8) and (9); thus, the image frame number $N_{F}$ and the star spot number $N_{S}$ contributed equally to the performance of the self-calibration. Therefore, the image frame number $N_{F}$ should be set similarly to the star spot number $N_{S}$ in the simulation experiment. After careful consideration, the number of image frames involved in the calculation was set to $N_{F}=10–100$. Finally, $N_{C}=100,000$ rounds of repeated simulations were conducted for each combination of parameters $\left(\sigma_{S},N_{S},N_{F}\right)$. 

Considering the estimation results of the intrinsic parameters in the kth simulation $\left({\hat{x}}_{o,k},{\hat{y}}_{o,k},{\hat{f}}_{k}\right)$ and their true values $\left(x_{o},y_{o},f\right)$, the bias evaluation parameters of the intrinsic parameters were defined as follows:

(14) $$\{\begin{array}{c} x_{o,bias}=\frac{1}{N_{C}}\underset{k}{\sum }{\hat{x}}_{o,k}-x_{o}\\ y_{o,bias}=\frac{1}{N_{C}}\underset{k}{\sum }{\hat{y}}_{o,k}-y_{o}\\ f_{bias}=\frac{1}{N_{C}}\underset{k}{\sum }{\hat{f}}_{k}-f\; \end{array},$$

and the total error evaluation parameters of the intrinsic parameters were defined by

(15) $$\{\begin{array}{c} x_{o,error}=\sqrt{\frac{1}{N_{C}-1}\underset{k}{\sum }{\left({\hat{x}}_{o,k}-x_{o}\right)}^{2}}\\ y_{o,error}=\sqrt{\frac{1}{N_{C}-1}\underset{k}{\sum }{\left({\hat{y}}_{o,k}-y_{o}\right)}^{2}}\\ f_{error}=\sqrt{\frac{1}{N_{C}-1}\underset{k}{\sum }{\left({\hat{f}}_{k}-f\right)}^{2}}\; \end{array}.$$

In Equation (15), the total error of the intrinsic parameters includes bias and random errors.

First, the convergence processes of different on-orbit calibration algorithms were analyzed using different values of the parameter λ, and the other parameters were set as follows: the number of image frames involved in the calculation was set to $N_{F}=100$, the star spot extraction error was set to $\sigma_{S}=0.10$ pixel, and the number of star spots was set to $N_{S}=20$. 

The initial values of the intrinsic parameters of the star sensor were set as follows: the position of the principal point of the optical system was the (1000, 1000) pixel, the focal length was 20 mm due to the a priori focus estimated from the ground laboratory calibration, and the general prior accuracy of the focus was higher than 10%. 

**Figure 4:**
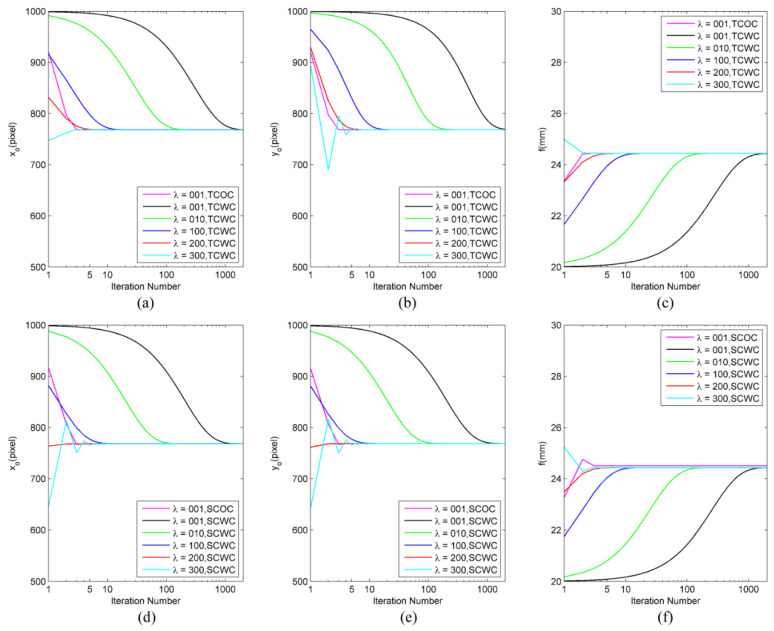
The convergence curves obtained by different calculation methods; the number of iterations and the value of scale parameter λ were used to calculate intrinsic parameters using a typical set of simulation data (the parameter settings were as follows: the number of image frames was $N_{F}=100$, the star spot extraction error was $\sigma_{S}=0.10$ pixel, the number of star spots was $N_{S}=20$, the initial position of the principal point of the optical system was (1000, 1000) pixel, and the focal length was 20 mm): (**a**–**c**) TCOC and TCWC; (**d**–**f**) SCOC and SCWC.

The convergence results of the intrinsic parameters were obtained by setting different iteration numbers and values for the scale parameter λ, as shown in Figure 4. After the optical path constraint was introduced (refer to Equation (9)), the convergence speed of the nonlinear least square method decreased, and the solution process required more iterations. When all four algorithms used the default value of λ (which was one), the SCOC and TCOC required five iterations to achieve convergence, whereas the SCWC and TCWC required approximately 1000 iterations. As the Jacobi matrix of $w_{mi}$ rather than the scale parameter λ determined the final convergence result of the nonlinear least square method, as long as the iteration process of the four methods did not diverge, the final convergence result was independent of the λ value. In addition, the number of iterations required by the SCWC and TCWC to achieve convergence gradually decreased with the increase in the λ value. Under the condition of convergence without oscillation, the number of iterations required for convergence was inversely proportional to the value of λ. However, when the λ value was too large (e.g., λ=300), the convergence speed could not be accelerated further, and fluctuation and even divergence could occur. If the λ value was further increased (e.g., λ=500), iteration divergence could be observed in almost every simulation. In this study, the value range of λ was 100–200, which was appropriate for the SCWC and TCWC, allowing the SCWC and TCWC algorithms to converge in 5–10 iterations. For the convenience of comparing the four algorithms, in the following simulation experiments, the scale parameter was set to λ=200 and the maximum number of iterations was set to 10.

**Figure 5:**
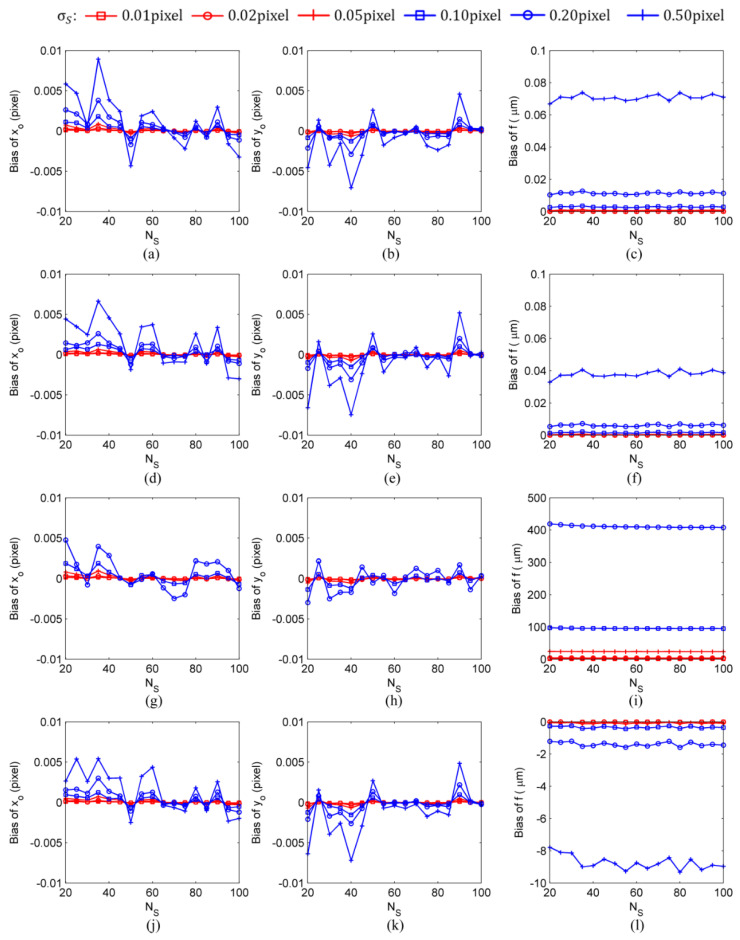
The intrinsic parameter biases calculated by different calibration methods under the conditions of a number of image frames of $N_{F}=100$, a star spot extraction error of $\sigma_{S}=0.01\;\mathrm{pixel}–0.50$ pixel, and a number of star spots of $N_{S}=20–100$: (**a**–**c**) TCOC; (**d**–**f**) TCWC; (**g**–**i**) SCOC; (**j**–**l**) SCWC.

**Figure 6:**
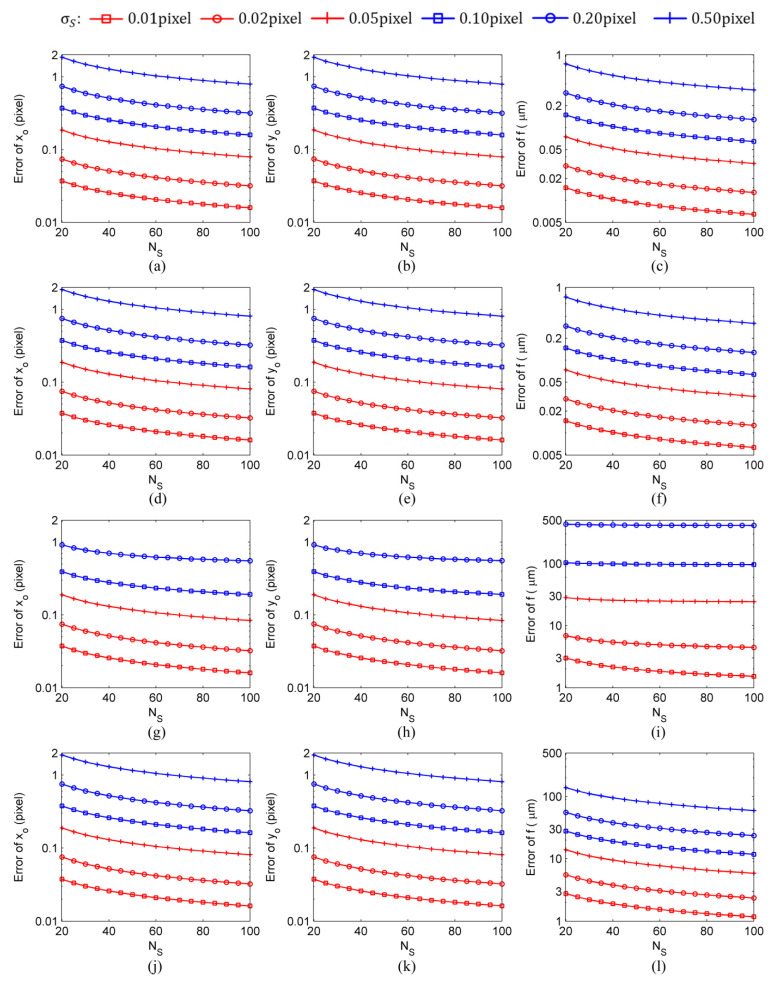
The intrinsic parameter total errors of different calibration methods under the conditions of a number of image frames of $N_{F}=100$, a star spot extraction error of $\sigma_{S}=0.01\mathrm{pixel}–0.50$ pixel, and a number of star spots of $N_{S}=20–100$: (**a**–**c**) TCOC; (**d**–**f**) TCWC; (**g**–**i**) SCOC; (**j**–**l**) SCWC.

**Figure 7:**
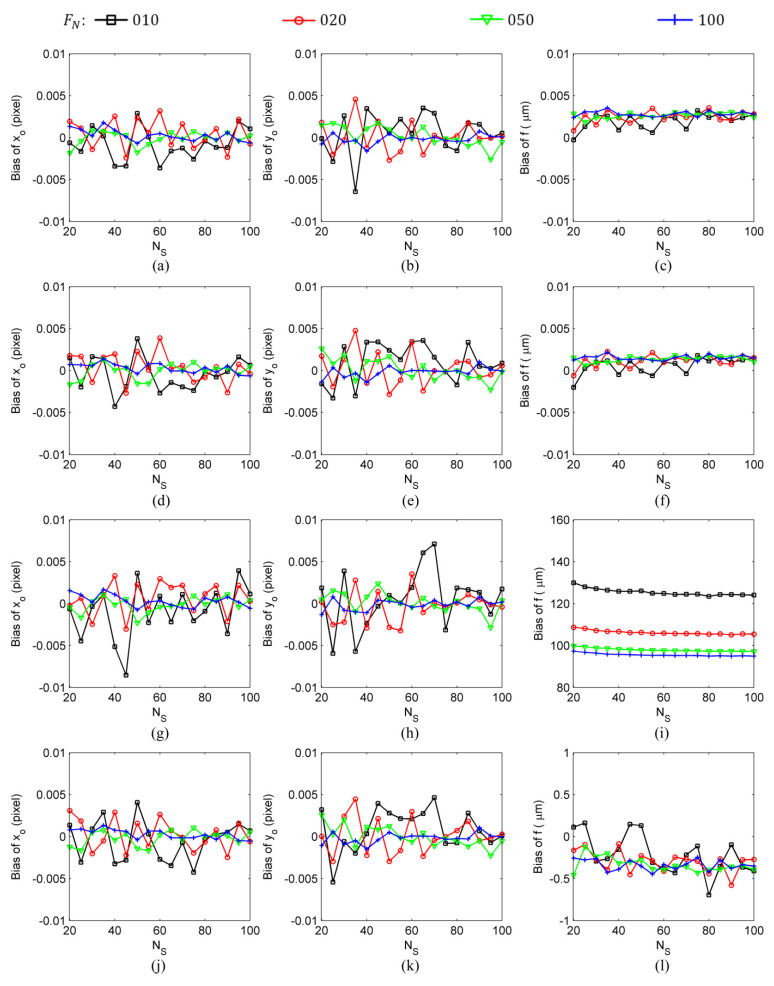
The intrinsic parameter biases obtained by different calibration methods under the conditions of a star spot extraction error of $\sigma_{S}=0.10$ pixel, a number of image frames of $N_{F}=10–100$, and a number of star spots of $N_{S}=20–100$: (**a**–**c**) TCOC; (**d**–**f**) TCWC; (**g**–**i**) SCOC; (**j**–**l**) SCWC.

**Figure 8:**
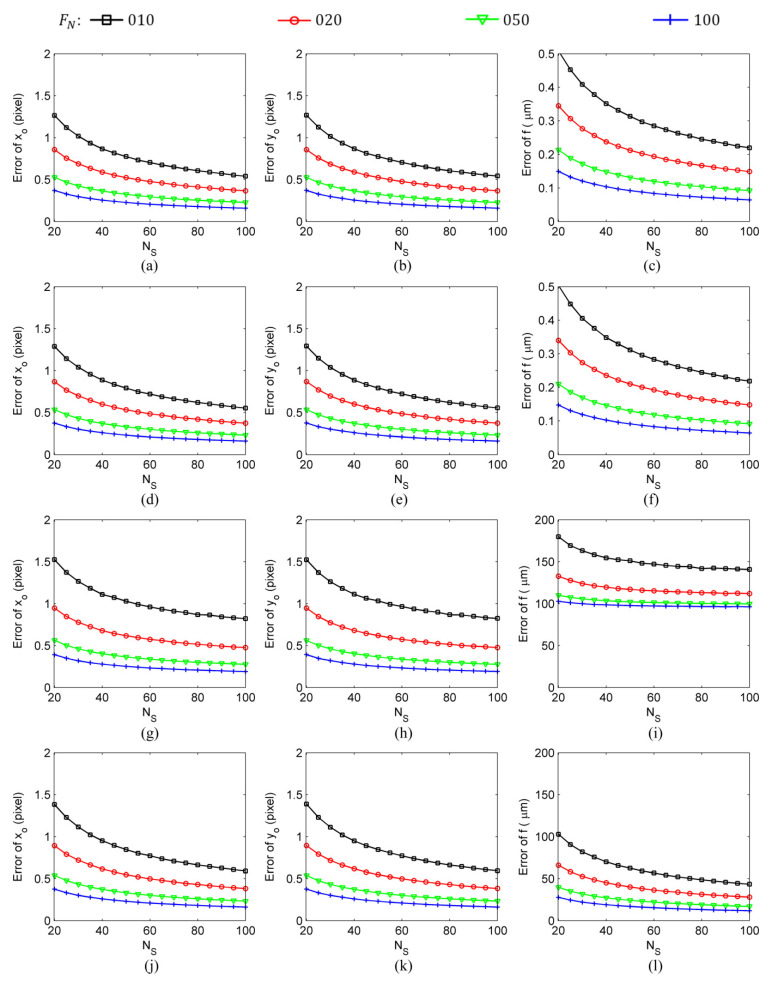
The results of the intrinsic parameter biases calculated by different calibration methods under the conditions of a star spot extraction error of $\sigma_{S}=0.10$ pixel, a number of image frames of $N_{F}=10–100$, and a number of star spots of $N_{S}=20–100$: (**a**–**c**) TCOC; (**d**–**f**) TCWC; (**g**–**i**) SCOC; (**j**–**l**) SCWC.

The calculation results shown in Figure 5 and Figure 6 were obtained with the following settings: the number of image frames used in the calculation was $N_{F}=100$, the star spot extraction error was $\sigma_{S}=0.01\;\mathrm{pixel}–0.50\;\mathrm{pixel}$, and the number of star spots was $N_{S}=20–100$. Finally, to determine the influence of the number of image frames on intrinsic parameter estimation errors, the calculation results were obtained under the typical settings of a star spot extraction error of $\sigma_{S}=0.10$ pixel, a number of image frames of $N_{F}=10–100$, and a number of star spots of $N_{S}=20–100$, as shown in Figure 7 and Figure 8.

The results of the simulation experiments showed that there was no bias in the estimation results of the principal point $\left(x_{o},y_{o}\right)$ obtained by the four methods. However, there were biases in the estimation results of the focal length calculated by the four methods. Compared to the calibration methods with constraint, the calibration methods without constraint could significantly reduce the bias of the focal length estimation results. In particular, the self-calibration methods could reduce the focal length bias by two orders of magnitude. In the simulation experiment conducted under the settings of a star spot extraction error of $\sigma_{S}=0.50$ pixel, a number of image frames of $N_{F}=100$, and a number of star spots of $N_{S}=20$ (refer to Figure 5), the SCOC obtained $f_{bias}>500\;\mu m$, the SCWC achieved $f_{bias}=-8.6\;\mu m$, the TCOC had $f_{bias}=0.07\;\mu m$, and the TCWC yielded $f_{bias}=0.04\;\mu m$. As for the self-calibration methods, when the star spot extraction error of $\sigma_{S}\geq 0.20$ pixel was not subjected to constraint, the estimated values of the intrinsic parameters did not converge. For instance, at $\sigma_{S}=0.5$ pixel, the bias of the focal length value calculated by the SCOC was larger than 500 μm. Even when the star spot extraction error decreased to $\sigma_{S}=0.2$ pixel, the bias $f_{bias}$ of the focal length value calculated by the SCOC was still larger than 400 μm, and the total focal length error $f_{error}$ was close to 500 μm, as shown in Figure 6; this indicated that the bias of the calculated focal length accounted for more than 80% of the total focal length error. In comparison, when the star spot extraction error was $\sigma_{S}=0.2$ pixel, the bias of the focal length value calculated by the SCWC was $f_{bias}=-1.2\;\mu m$, and the total focal length error was $f_{error}=55\;\mu m$; this indicated that the bias of the calculated focal length accounted for less than 3% of the total focal length error. Therefore, it could be concluded that it is necessary to add a constraint to the estimation process of the intrinsic parameters of a star sensor conducted by a self-calibration method (refer to Equation (9)).

Compared with the self-calibration methods that use only information in an image space, traditional calibration methods, due to the availability of the coordinate information of the stars on the celestial sphere, can use the information in both the object space and image space and, thus, can achieve higher estimation accuracy (two to three orders of magnitude higher) for the focal length of the star sensor. However, the two types of calibration methods show no significant difference in their estimation of the principal point. In the simulation experiment conducted under the settings of a star spot extraction error of $\sigma_{S}=0.10$ pixel, a number of image frames of $N_{F}=100$, and a number of star spots of $N_{S}=20$ (refer to Figure 8), the SCOC obtained $f_{error}=103\;\mu m$, $x_{0,error}=y_{0,error}=0.37$ pixel; the SCWC achieved $f_{error}=28\;\mu m$, $x_{0,error}=y_{0,error}=0.37$ pixel; the TCOC had $f_{error}=0.15\;\mu m$, $x_{0,error}=y_{0,error}=0.37$ pixel; and, finally, the TCWC yielded $f_{error}=0.15\;\mu m$, $x_{0,error}=y_{0,error}=0.37$ pixel. A comparison of the results presented in Figure 7 and Figure 8 reveals that, among the intrinsic parameter estimation errors of the traditional calibration methods, the bias was about two orders of magnitude smaller than the random error. Since the bias was not the main part of the intrinsic parameter estimation error, it could be ignored. Therefore, the bias of intrinsic parameter estimation is not an issue to be considered in research on traditional on-orbit calibration methods.

For traditional on-orbit calibration methods, it is natural to extend the pinhole imaging model of a star sensor to a high-order distortion model [4,20], but this is not the case for self-calibration methods. Since the estimation accuracy of the intrinsic parameters obtained by self-calibration methods is far lower than that of traditional on-orbit calibration methods, it is a great challenge for self-calibration methods to extend the pinhole imaging model of a star sensor to a high-order distortion model. Therefore, improving the accuracy of self-calibration algorithms and extending the pinhole imaging model to a high-order distortion model are still challenges that should be addressed in future research.

A further algorithm complexity analysis showed that, due to the use of interframe information in self-calibration, the number of measurement equation sets was $O\left(N_{F}^{2}N_{S}^{2}\right)$, which was much higher than the number for traditional calibration, $O\left(N_{F}N_{S}^{2}\right)$. When the calibration was deployed on the hardware of a satellite, the limitations of storage, reliability, and power consumption posed additional challenges to the operation of the self-calibration algorithm.

Compared to existing calibration methods, the proposed self-calibration demonstrated no advantages in terms of estimation accuracy and algorithm complexity. Thus, there arises a question of whether research on this type of algorithm is still meaningful. The answer is yes because, in traditional on-orbit calibration based on star coordinates in the celestial sphere (i.e., star identification), when star identification cannot be performed before the intrinsic parameter determination of a star sensor, the only choice is to determine intrinsic parameters based on image-space information (i.e., self-calibration) before star identification.

The primary factor to consider in star map identification for the pinhole imaging model is the focal length or the nominal pixel scale in the sky. In our experiments, when the parameters were set to their typical values (i.e., the typical star spot extraction error was $\sigma_{S}=0.10$ pixel, the number of image frames was $N_{F}=100$, and the number of star spots was $N_{S}=20$), the SCWC achieved $f_{error}=28\;\mu m$, its relative error was 0.11%, and its direction error corresponding to the edge of the field of view was approximately 40″. For a typical star sensor with a sensitive magnitude error of less than 0.5 Mv, the full-sky star identification probability of the traditional star map identification algorithm [10] was still better than 98%. Thus, for a star sensor with typical parameters, the intrinsic parameters determined by on-orbit calibration could be directly used in a traditional star map identification algorithm. After star map identification, the traditional on-orbit calibration method for star sensors could be used to improve the intrinsic parameter estimation accuracy of star sensors further for high-precision attitude determination. 

Furthermore, the extraction error of star spots is an important factor affecting the estimation accuracy of self-calibration algorithms. Star sensors use the centroid of star spots as their image coordinates, making it challenging to achieve a positional accuracy of 0.01 pixel. For instance, AST-301 [24] has a centroid extraction error of 0.02 pixels, which is a representative of extremely high-precision star sensors. In recent years, there have been certain developments, such as interferometric imaging [25] and point spread function (PSF) reconstruction [26], where pixel subdivision accuracy exceeds 0.001 pixels. Finally, the intrinsic parameters determined by on-orbit calibration with high-accuracy pixel subdivision can be directly used in the high-precision attitude determination of star sensors.

## 6. Conclusions

This paper proposes an on-orbit self-calibration method for star sensors with the aim of addressing the chicken-and-egg problem of the star identification and intrinsic parameter determination of star sensors during their on-orbit operation. Unlike traditional on-orbit calibration methods, which perform dimensionless star map identification before determining intrinsic parameters, the proposed method does not require star identification. First, a set of equations for the intrinsic parameters of a star sensor are established based on the invariance of the interstar angle of a star pair between image frames; these equations do not require the true value of the interstar angle of the star pair. Then, the constant constraint of the optical path from the star spot to the center of the star sensor optical system is defined to reduce the biased estimation in self-calibration. Finally, a scaled nonlinear least square method is developed to solve the self-calibration equations to accelerate iteration convergence. The simulation and analysis results show that the bias of the focal length estimation in on-orbit self-calibration with constraint is two orders of magnitude smaller than that in on-orbit self-calibration without constraint. In addition, convergence can be achieved in 10 iterations when the scaled nonlinear least square method is used to solve the self-calibration equations. Further, on-orbit calibration can be accomplished using a star sensor directly, without prior star identification, which addresses certain problems in the dimensionless star map identification method, such as high computational complexity and the need for a large database.

In this study, the key points of distant static objects in multiple image frames are obtained by rotating a camera, which can be approximated as the continuous imaging of stars using a star sensor. Theoretically, the proposed calibration model and numerical solution method can be used to calibrate common cameras by observing distant static objects with unknown geometric dimensions. Also, the accuracy of self-calibration algorithms is much lower than that of traditional camera calibration methods, so it is of great importance to improve the accuracy of self-calibration algorithms. Therefore, in the future, self-calibration methods could be further analyzed while paying special attention to the above two issues.

## Data Availability

All code, data, and materials included in this research are available upon request from the corresponding author.
